# Sialyl-Tn in Cancer: (How) Did We Miss the Target?

**DOI:** 10.3390/biom2040435

**Published:** 2012-10-11

**Authors:** Sylvain Julien, Paula A. Videira, Philippe Delannoy

**Affiliations:** 1 Structural and Functional Glycobiology Unit, UMR CNRS 8576, University of Sciences and Technologies of Lille, 59655 Villeneuve d’Ascq, France; Email: philippe.delannoy@univ-lille1.fr; 2 CEDOC, Departamento de Imunologia, Faculdade de Ciências Médicas, Universidade Nova de Lisboa, 1169-056 Lisboa, Portugal; Email: paula.videira@fcm.unl.pt

**Keywords:** Sialyl-Tn antigen, *O*-glycan, ST6GalNAc I, Theratope, cancer immunotherapy

## Abstract

Sialyl-Tn antigen (STn) is a short *O*-glycan containing a sialic acid residue α2,6-linked to GalNAcα-*O*-Ser/Thr. The biosynthesis of STn is mediated by a specific sialyltransferase termed ST6GalNAc I, which competes with *O*-glycans elongating glycosyltransferases and prevents cancer cells from exhibiting longer *O*-glycans. While weakly expressed by fetal and normal adult tissues, STn is expressed by more than 80% of human carcinomas and in all cases, STn detection is associated with adverse outcome and decreased overall survival for the patients. Because of its pan-carcinoma expression associated with an adverse outcome, an anti-cancer vaccine, named Theratope, has been designed towards the STn epitope. In spite of the great enthusiasm around this immunotherapy, Theratope failed on Phase III clinical trial. However, *in lieu* of missing this target, one should consider to revise the Theratope design and the actual facts. In this review, we highlight the many lessons that can be learned from this failure from the immunological standpoint, as well as from the drug design and formulation and patient selection. Moreover, an irrefutable knowledge is arising from novel immunotherapies targeting other carbohydrate antigens and STn carrier proteins, such as MUC1, that will warrantee the future development of more successful anti-STn immunotherapy strategies.

## 1. Introduction

Sialyl-Tn (STn) is a carbohydrate antigen discovered as a cancer marker in the early 80s. Detected in virtually all epithelial cancers investigated, STn has known a 20 years long golden age as a research topic with two main focuses. The first focus, until about the mid 90s, was the value of STn as a marker for diagnosis and subsequently prognosis in cancer. The second focus was the targeting of STn by state-of-the-art immunotherapy strategies to treat cancers, notably breast cancer. On this topic, a very enthusiastic literature spanned from the mid 90s to 2005, the year when STn was somewhat crushed and buried under the failure of the Phase III clinical trial of the Theratope.

Writing this review, we have tried to provide the reader with the most comprehensive vision of STn in cancer, from its molecular synthesis to its possibly underestimated usefulness as a therapeutic target. A substantial part of this manuscript is dedicated to the expression of STn across various types of cancers in order to discuss, in depth, the therapeutic strategies, past and future, targeting STn to treat cancer.

## 2. Sialyl-Tn Structure and Biosynthesis

**Figure 1:**
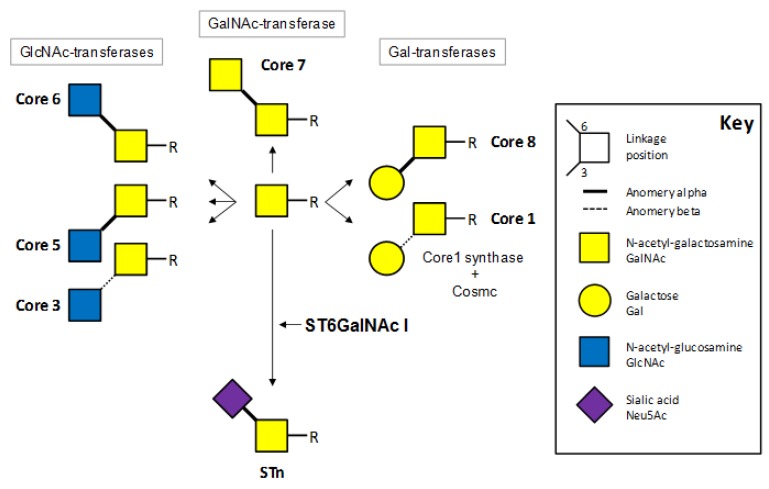
The initial steps of the *O*-glycan biosynthesis showing cores and sialyl-Tn (STn) competition for the initial GalNAc.

STn (Neu5Acα2-6GalNAcα-*O*-Ser/Thr), also referred to as CD175s by the “cluster of differentiation” nomenclature, is the simplest sialylated mucin-type *O*-glycan. STn is a disaccharide formed of one residue of *N*-acetyl-galactosamine (GalNAc) alpha-*O*-linked to a serine or a threonine residue, and substituted by a sialic acid (Neu5Ac in human) on carbon 6. This sialylation prevents the formation of various core structures otherwise found in mucin-type *O*-glycans (Figure 1).

Biosynthesis of *O*-glycans is a step-by-step process that occurs in the Golgi apparatus. *O*-glycans are synthesised by the sequential action of several glycosyltransferases, each transferring a monosaccharide [e.g., GalNAc, Neu5Ac, galactose (Gal), *N*-acetyl-glucosamine (GlcNAc)] from a donor nucleotide-sugar (e.g., UDP-GalNAc or CMP-Neu5Ac) to an acceptor that is the glycan being synthesized. Glycosyltransferases are membrane-bound enzymes of which the level of expression, substrate specificity and localisation in Golgi compartments are responsible for the pattern of *O*-glycans expressed in a given cell or carried by a given glycoprotein. Furthermore, because several distinct enzymes may compete for the same acceptor structure, their relative expression and activities are decisive for the structures expressed in each cell. Consequently, STn expression depends on the expression of polypeptide-GalNAc-transferase and sialyltransferase activities. Similarly, STn expression might be promoted by the weak expression or activity of enzymes competing with sialyltransferases that are the Gal-, GalNAc- and GlcNAc-transferases that synthesize *O*-glycan ‘cores’ (Figure 1).

Polypeptide GalNAc-transferases constitute a family of 20 enzymes, all catalyzing the transfer of a GalNAc on the protein backbone. This diversity allows a finely tuned control of the initiation of *O*-glycosylation in a cell-specific and, possibly, a protein-specific manner. As the GalNAc is necessary for the transfer of sialic acid to create STn structure, one can say that the efficient GalNAc-transferase activity is crucial for STn expression. Logically, cells lacking these activities are therefore unable to express STn or any other GalNAc-based *O*-glycans, as observed in the testis for spermatogonia and Sertoli cells [1]. However, to date, there is no demonstration that the quantity or the quality (e.g., clustering or specific sites) of *O*-GalNAcylation of proteins affects the subsequent sialylation of GalNAc residues.

Sialylation of single *O*-GalNAc has been shown to be performed *in vitro* by two members of the sialyltransferase family, namely ST6GalNAc I and ST6GalNAc II [2,3]. However, studies using cells transfected by either one of these enzymes have demonstrated that in a cellular context, only ST6GalNAc I is able to create STn structures as recognized by anti-STn antibodies [2,4]. Furthermore, ST6GalNAc I over-expression was shown to correlate with STn expression in gastric and breast tumors confirming the crucial role of ST6GalNAc I in STn biosynthesis [4,5]. Indeed, over-expression of ST6GalNAc I is able to compete with *O*-glycan cores biosynthesis as shown in MDA-MB-231 breast cancer cells where stable expression of ST6GalNAc I converted 22% of Core 1-based *O*-glycans carried by MUC1 mucin into STn [6]. 

ST6GalNAc I activity can be eased by a weak activity of competing core-synthases. One such mechanism has been discovered by Cummings *et al*. who reported the existence of a chaperone protein for the Core 1 synthase (core 1 β1,3-galactosyltransferase) designated Cosmc (core 1 β3-Gal-T-specific molecular chaperone) [7]. Mutations and loss of heterozygosity of the *COSMC* gene were described in STn-positive melanoma and colon cancer cell lines, as well as in tissue samples from two STn-positive cervical cancers [8]. However, extensive studies of *COSMC* mutations in breast or colon cancers showed that these events were rare and could only account for some of the cases of STn expression in cancers [9].

Thus, STn expression in cancer is most probably due to over-expression of ST6GalNAc I, with enhancing effects of increased synthesis of precursors (*i.e*., increased transfer of *O*-GalNAc) and decreased competition (*i.e*., decreased core-synthesis).

## 3. STn Immunodetection in Tissues and Tumors

### 3.1. Anti-STn Antibodies

Many anti-STn antibodies have been developed since the mid 80s (see Table 1 for the most used ones), each using different immunogens and displaying subtle differences in their affinity for STn antigen. These differences may account for conflicting results when comparing several studies reporting STn expression.

**Table 1:** Most used anti-STn monoclonal antibodies.

Monoclonal antibody	Immunogen	Isotype	Specificity	Ref.
**B72.3**	Membrane fraction of breast cancer metastasis	Mouse IgG1	Clustered STn bound to serine. Cross reacts with Tn clusters	[10,11]
**MLS102**	LS 180 colonic cancer cells	Mouse IgG	Clustered STn, independently of the linkage to the peptide backbone.	[12,13]
**TKH2**	Ovine submaxillary mucin	Mouse IgG	Monomeric STn	[14,15]
**HB-STn1**	Ovine submaxillary mucin	Mouse IgG1		[16]

### 3.2. Expression in Normal Tissues

#### 3.2.1. Fetal Tissues

There are a few reports of STn detection in fetal organs such as esophagus and stomach [17,18], pancreas [12], colon (goblet cells) [17,19], lung [18], mammary gland [20], and gonadal tissues from fetuses of both sexes [1,18]. Furthermore, STn was detected in meconium and amniotic fluids [21] suggesting a relation to digestive tract secretions. However, nothing is known about the biological role of STn during embryonic development. 

#### 3.2.2. Adult Healthy Tissues

STn expression was rarely systematically screened throughout normal adult tissues [14,17,22]. However, many studies focusing on STn expression in cancer also reported staining performed on normal healthy tissues used as control. STn expression seems to be closely related to the upper digestive tract, since it was detected in salivary glands, esophagus and stomach. This expression is finely regulated and restricted to some specific cell types such as squamous cells in salivary glands and esophagus [17,22,23], mucous acini in the submandibular gland and submucosae of the vestibular fold and soft palate [24], and parietal and goblet cells in the stomach [25,26,27]. In the colon, STn was visible in colonocytes only and only after the removal of *O*-acetyl groups by saponification [14,15,28]. STn was also detected in bile ducts [17,22] but never detected in normal hepatocytes [22,29]. Likewise, STn antigen was never found in normal pancreas [10,14,17,22,30,31,32]. 

Within the urogenital tract, STn was detected in uterine and cervix cells and always with a membranous apical staining [22,33,34]. In contrast, normal ovarian epithelia were always found to be STn negative [35,36,37] Some testis cells were occasionally found to be STn positive such as Leydig cells or interstitial cells [1,14,22]. Finally, STn was found at the apical membrane of the ciliated cells of the lung [17,22,38] and at the apical side of the ductal epithelium in normal breast tissue [39,40,41]. 

Although quantified data are not properly available, overall, the authors reported STn expression in normal tissues to be rare and/or low compared to cancer tissues. Reports stating that STn expression is restricted to cell types specialized in secretion, apical surface of secreting cells or even content of lumen of secretory structure all suggest that sparse expression of STn in normal tissue relates to external fluids of the body. One may thus hypothesize that STn is unlikely to be naturally present in the bloodstream or the lymph. 

### 3.3. STn Expression in Cancers

**Figure 2:**
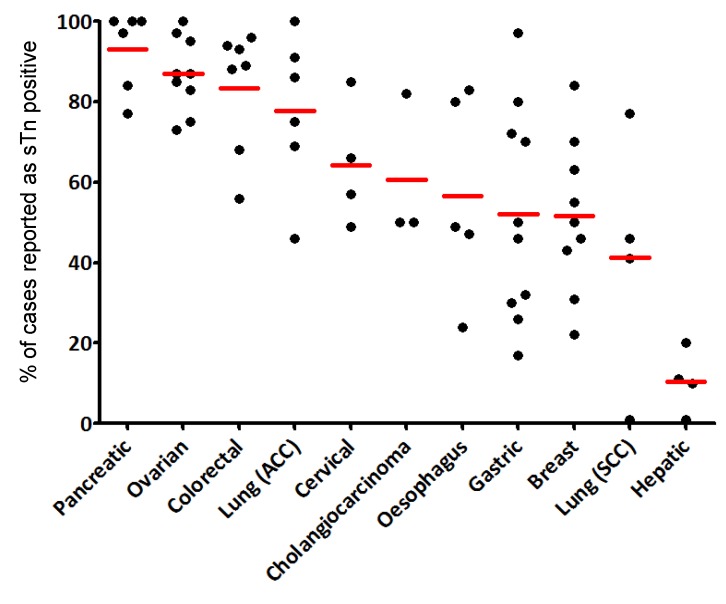
STn frequency in the most studied cancers. Each dot represents a report (see Figure 3 for references). The red bar is the mean of the percentage of STn positive cases.

From the characterization of the first anti-STn monoclonal antibodies, it was rapidly discovered that STn antigen was over-expressed in cancer cells compared to the matching healthy cells; hence STn was described as an onco-fetal antigen. STn neo-expression or over-expression was reported in many epithelial cancers with highest frequencies in pancreas, colorectal and ovarian cancers (Figure 2). STn was therefore considered as a good tumor marker of carcinogenesis and potentially useful for diagnosis.

**Figure 3:**
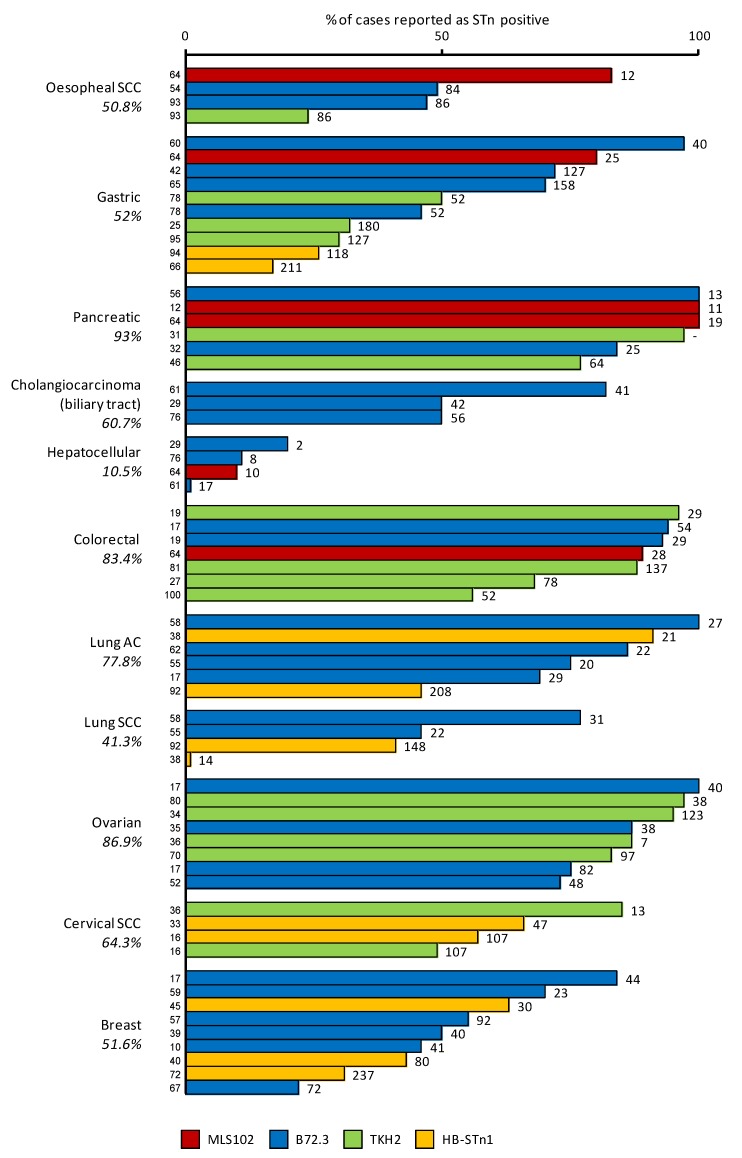
STn frequency in various carcinomas. The diagram shows the percentage of STn positive cases detected in various carcinomas using different anti-STn mAbs. The average percentage of positive cases is indicated below the type of cancer. The numbers on the top of the bars indicate the number of samples used for each study. References are indicated at the base of each bar, including [56,57,58,59,60,61,62] that are not quoted elsewhere in the text. SCC: Squamous cell carcinoma, AC: adenocarcinoma.

However, we invite the reader to keep in mind that many parameters may account for the high variability in the frequencies reported by different authors. Amongst those factors are the distinct antibodies used, the protocol of staining, the method of scoring, the size of the cohort of the samples and the heterogeneity of cancer subtypes included in the respective studies. Some of these elements are included in Figure 3 for a more comprehensive reading.

#### 3.3.1. Early in Carcinogenesis

STn was reported to be over-expressed in several epithelial benign lesions considered to be potential precursors of cancers, such as esophageal dysplastic squamous epithelia [23], gastric intestinal metaplasia [42], colonic moderate dysplasia [43], lung atypical adematous hyperplasia [38], or breast ductal hyperplasia and apocrine metaplasia [40,44,45]. In these different cases, STn over-expression was primarily observed at the apical or luminal cell surface as well as in the corresponding healthy tissues. More unexpectedly, STn was also reported in benign lesions in pancreas [46] and ovaries [35,36,37], two tissues that are devoid of STn expression in the healthy state. Notably, STn staining was observed in pancreatic intraepithelial neoplasia stage III (PanIN3), the last histological grade relevant to benign tumor before the tumor becomes invasive, but not in earlier stages [46,47]. All together, these observations suggest that STn over-expression occurs earlier in carcinogenesis in tissues that normally express the antigen than in the others. 

There is a body of evidence linking STn expression to inflammatory diseases of the stomach (gastritis) or the colon (ulcerative colitis and Crohn’s colitis). In gastritis, STn was detected in 50–100% of the cases [48,49] and it appeared to be independent on the presence of *Helicobacter pylori*, a common bacterial parasite of the stomach [48]. In ulcerative colitis, de-*O*-acetylated STn was shown to be an independent marker of the dysplasia-carcinoma sequence [50]. De-*O*-acetylated STn was also detected in 44% of the cases of Crohn’s colitis, another inflammatory disease associated with colon cancer risks [51]. These reports suggest that STn expression might be regulated by inflammatory signals, such as γ-interferon [51], in pre-malignant lesions of these two organs. Whether or not such signals could trigger STn expression in other organs remains to be determined.

#### 3.3.2. Cytological Types of Carcinomas

Epithelial cancers, or carcinomas, are divided into two major types depending on the cytology of the cancer cells: adenocarcinomas and squamous cell carcinomas. Adenocarcinomas (AC) arise from glandular epithelia and often secrete gland related molecules (e.g., mucins). When these secretions are abundant, AC are classified as mucinous carcinomas. By contrast, squamous cell carcinomas (SCC) are formed of thin, flat and poorly secreting cells. 

Cancers arising in the pancreas, ovary, colon, breast, stomach and liver are more likely to be AC, whereas cancers of the cervix or esophagus are more often SCC. Non-small cell lung cancers may be either AC or SCC and arise from a common precursor cell located in the basal bronchial epithelium. 

The cellular pattern of STn expression is different according to the cancer cell morphology. Mucinous cancer cells are stained on the whole cell membrane, focally in the cytoplasm (perinuclear area, presumably Golgi) and sometimes in extracellular compartments [19,39,52]. This indicates that STn is carried by both membrane bound and secreted glycoproteins such as mucins. The role of mucins as STn carriers in mucinous cancers has recently been confirmed using the Proximity Ligation Assay [53]. Squamous cancer cells are mainly and intensively stained in the cytoplasm and sometimes on the cell membrane [23,52,54]. This supports the idea that STn antigen is carried by a diversity of glycoproteins differently expressed and/or processed according to the cell type. Furthermore, it appears that STn is more often detected in AC than in SCC in both cervix [16] and lung cancers [38,55]. Because STn seems to be carried by different glycoproteins and in different compartments in AC and SCC, its putative biological function in cancer development might be different in these tumor types.

#### 3.3.3. Cell Differentiation

Loss of cell differentiation, often participating in a high histological grade classification, was reported to qualitatively modulate the STn expression. In colonic poorly differentiated cells, STn cytoplasm staining is more frequent than in well differentiated cells [19,63]. There is also a redistribution of antigen expression to the whole cell membrane, including the basolateral surface [43,63]. Loss of differentiation was reported to be associated with a decreased frequency of STn positive cases in pancreatic [46] and colorectal cancers [43]. Inversely, a tendency towards increased STn expression frequency in poorly differentiated cells was observed in liver and bladder cancers [29]. Finally, the histological grade does not seem to affect STn expression in gastric [25,64,65] or breast cancer [66,67]. All these observations are subjected to differences in histological grading of each tumor type, which may reflect different biological events for the cancer cells, depending on the organ considered. However, using nasopharyngeal cell lines, Lin *et al*. recently showed that STn expression was associated with epithelial to mesenchymal transition [68], a loss of differentiation that is an important milestone towards cancer metastasis.

#### 3.3.4. Heterogeneity within Tumors

STn expression is generally reported to be heterogenous in tumors with proportions of STn-positive cells ranging from 5% to 100% (rarely 100%). This phenomenon is consistent whatever the origin of the tumor: stomach [25,26], colon [43], ovary [52,69], cervix [33] and breast [39,70,71].

Various clinical features were correlated with the degree of heterogeneity of STn expression. For example, Federici *et al.* reported that ovarian mucinous cancers were more likely to be uniformly stained than ovarian serous cancers [52]. Lopez-Ferrer *et al*. also observed that the percentage of STn positive cells was higher in lung AC (34%) than in SCC (9%) [55]. In gastric cancers, an increased percentage of STn positive cells was correlated to deeper invasion and advanced stage [25]. Flucke *et al*. reported that patients with more than 35% of stained cells in their esophageal SCC (25% of cases) had a decreased overall survival, compared to the low expressing group (<35% positive cells) [54]. 

In ovarian cancers, Davidson *et al*. observed that STn expression was sometimes more intense at the invasive front of the tumor and that the percentage of STn positive cells was higher in effusions than in the matching primary tumors [69]. We reported a similar pattern of expression at the periphery of the tumors in a model of breast cancer cells injected as xenograft in SCID mice [6]. Because in this model the cells were all derived from a selected clonal population, the heterogeneity of STn was assumed to be related to the expression of the protein(s) that carried the glycan. In other words, STn expression could be regulated in the tumor, *via* the regulation of its carrier(s). All together, these observations suggest that STn expression could be correlated with the invasive and aggressive potential of epithelial cancer cells, when expressed at the right time and right place. 

#### 3.3.5. Association with Invasiveness

For a majority of the authors, STn expression detected in tissue and/or sera samples of patients with gastric cancers was correlated with depth of invasion [25,42,72,73,74,75,76], lymph vessel and venous invasion [65,72,73] and peritoneal dissemination [25,73]. Ikeda *et al*. reported that stromal STn detection was associated with peritoneal dissemination [77]. In accordance, Ozaki *et al*. recently reported that STn expression increased peritoneal metastasis in a model of human gastric cell lines transplanted in nude mice [78]. 

In samples from ovarian cancer patients, STn positive cells were more frequently observed at the invasion front of tumors and in peritoneal and pleural effusions, but less often in metastatic lesions than in primary tumors [69,79]. These results suggest that in ovarian cancers, STn enhances the dissemination of cells, facilitating primary tumor/effusion transition, but does not improve the settlement of metastatic cells in distant organs. 

However, in colorectal cancers, STn expression was reported not to be correlated with depth of invasion [15,80]. Similarly, Schmitt *et al*. have reported that breast ductal invasive carcinomas are less frequently STn positive than ductal carcinomas *in situ* [40]. Thus, sparse data for colorectal and breast cancer suggest that the effect of STn expression on the invasiveness of tumor cells might be an organ-specific phenomenon. However, *in vitro* characterization of engineered breast cancer cell lines showed that STn expression induced a decreased adhesion, a decreased aggregation and an increased cell motility, all consistent with an increased invasiveness [6,81]. Whether or not these observations are relevant at the disease level for breast or colorectal cancers would require further investigations.

#### 3.3.6. Detection in Serum

Detection of tumor markers in serum is a simple, non-invasive and sensitive method for diagnosis or post surgery follow-up of the patients. This is particularly useful for the care of patients with cancers in deep organs (stomach, colon, pancreas, biliary tract, ovaries or cervix), which are often asymptomatic at the earlier stage. The presence of STn antigen in serum is due to important *O*-glycoprotein secretion or to cell shedding from tumors into the bloodstream, both requiring a large amount of cancer cells in the primary tumor. This critical tumor mass is usually found in advanced cancers, which are more likely to be of poor prognosis. 

A high level of STn (cut-offs ranging from 38 to 50 U/ml) was detected in sera of patients to various degrees, depending on the cancer type: twenty-eight to eighty-six percent in gastric cancers [72,73,82], 11%–28% in colorectal cancers [82,83], 40%–55% in pancreatic cancers [82,84], 25%–53% in biliary tract cancers [82,84], 29%–69% in ovarian cancers [36,85,86,87] and 15% in cervical cancers [36]. Unsurprisingly, a high level of serum STn was significantly associated with tumor size, lymph node, liver metastasis and advanced stages in gastric cancers [72,73,76]. Furthermore, high serum STn was found to be associated with a decreased overall survival of patients with gastric [72,88,89], colorectal [83,90] or ovarian [86,87] cancers. Thus, STn detection in serum is more useful for prognosis than for diagnosis and is usually considered as a poor prognosis marker.

#### 3.3.7. Prognosis Value of STn in Cancers

Because of its putative role in cancer cell invasion and spreading, there is a consensus to say that STn expression is associated with an adverse outcome, such as lymph node or distant metastasis and decreased overall survival of the patients. However, this may not be true for all types of cancers. 

**Figure 4:**
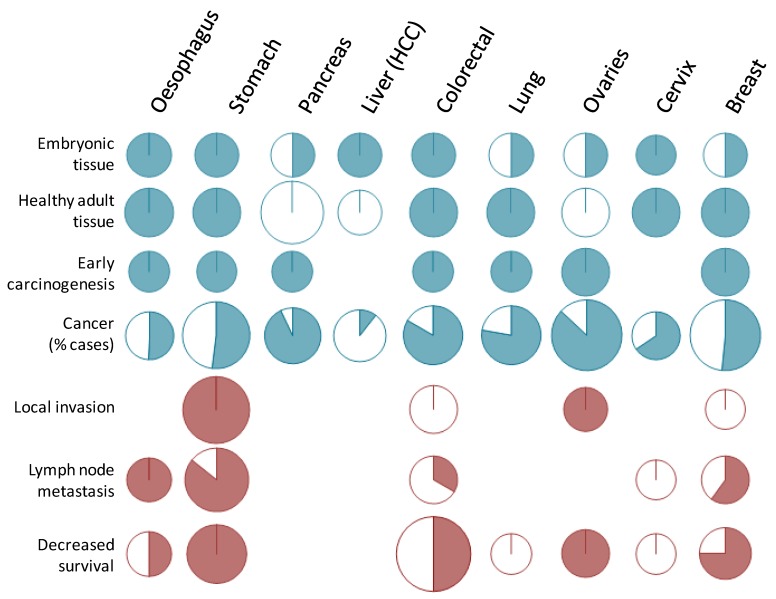
Graphic overview of STn expression in cancers. The picture represents the published papers reviewed herein sorted by cancer type. Top part: reports of positive (blue) or negative (white) expression in various tissues and cancer. For first and second lines, positive expression relates to a sparse and low expression as described in Section 3.2.1. and Section 3.2.2. In colorectal cancer, *O*-acetylated STn is considered to be a positive report since it requires the activity of ST6GalNAc I (see Chapter 2.). The forth line summarizes Figure 2 and Figure 3. Bottom part: reports assessing the correlation between STn expression and clinical features of cancers, with a significant correlation found in pink and no correlation found in white. Size of the circles represents the number of published reports (diameter calculated as 1 + 0.1 unit per publication).

For example, STn was not associated with lymph node invasion or overall survival in cervical cancer [16] or survival in lung cancer [91]. In esophageal cancers, Flucke *et al*. reported an association with decreased survival, disagreeing with Ikeda *et al*. while they both reported an absence of correlation with lymph node metastasis or TNM staging [54,92]. However, there are only few studies concerning these types of cancer, and extensive investigations might prove to be more insightful. Furthermore, there are no data available concerning the prognostic value of STn in pancreas or liver cancers. These gaps are probably due to the fact that STn expression is very frequent in pancreatic cancers (≈90%) and very rare in hepatic ones (≈10%) (Figure 2, Figure 3 and Figure 4), making the design of an unbiased cohort difficult for statistical analysis.

Nonetheless, as more studies are available we can draw an overall picture of the prognostic value of STn in gastric, colorectal, ovarian and breast cancers.

In gastric cancers, numerous studies reported a positive association of STn expression (in serum or primary tumors) with lymph node metastasis [25,73,75,88,89,93], peritoneal metastasis [25,73,77,88] and liver metastasis [73,88] with few contradicting reports [65]. Logically, many authors also reported a correlation between STn expression and decreased survival [26,65,72,75,88,94].

In colorectal cancers, few authors reported STn association with distant metastasis [95]. Reflecting this weak relationship of STn to invasiveness, the association of STn with decreased survival was reported by some authors [27,80,83,90,96] but contradicted by others [95,97,98,99]. 

STn association with poor prognosis is clearer in ovarian cancers since all authors concurringly reported a decreased survival for patients expressing high levels of STn in tissues or serum [35,86,87]. However, the way STn may affect survival is somewhat elusive, since STn may not be involved in the metastatic process of ovarian cancers [69].

Finally, STn over-expression was reported to correlate with negative estrogen receptor (ER) status in breast cancer [67,100], and ER-negative breast cancers are known to be more aggressive than ER-positive ones. However, one paper reported an association of STn with lymph node invasion [100] while two others disagreed [66,67]. Regarding survival, authors reported some association of STn with poor prognosis depending on various clinico-pathological parameters. For instance, Miles *et al*. reported a significant correlation of STn expression with decreased survival for patients with lymph node metastasis [71]. Kinney *et al*. concluded that STn was an independent predictor of recurrences [70]. Leivonen *et al*. reported a decreased survival of STn patients in the short-term (five years) but not in the long-term (>15 years) [67], whereas Imai *et al.* did not find any correlation at all [66]. 

So, from a general point of view, it seems that STn expression is mostly associated with an adverse outcome. However, the numerous discrepancies and subtleties reviewed above suggest that if STn exerts any biological function promoting cancer development, it occurs through various mechanisms depending on each cancer type or sub-type. 

## 4. Immunotherapy Targeting STn

The exploitation of the patient’s own immune system, i.e. immunotherapy, to control cancer growth rises as an attractive approach, offering the potential of enhancing the effects of conventional treatment such as post-surgery radiotherapy or chemotherapy, without substantially increasing toxicity. While boosting immune responses against cancer is obviously not sufficient to eradicate a solid tumor by itself, it might be crucial in the elimination of minimal residual disease or micrometastases following primary treatment or in the prevention of transformation from a *benign* to a precancerous *lesion*. 

There are several current immunotherapeutic approaches to treat cancer that have been approved for use in humans, such as cytokines (e.g., interleukin-2 [101]), which are able to modulate the leukocyte function, and monoclonal antibodies (e.g., Cetuximab), which recognize tumor antigens and hence mount a specific immune response against cells expressing them.

Vaccination or immunization is a promising immunotherapeutic approach that is being evaluated in a variety of different tumor types, targeting different antigens, in an attempt not only to boost anti-tumor immune responses, but also to develop immunologic memory, producing long-lived protection lasting for years or even decades. The side effects associated with immunization are mild compared with the conventional cytotoxic therapies. However, contrarily to microbial antigens, tumor-associated antigens may be considered self-antigens and the use of cancer vaccines may therefore easily result in enhanced activity of self-reactive lymphocytes and autoimmune responses. Thus, the designation of right target antigens is essential for successful tumor vaccination.

The finding of the STn antigen as a good tumor marker and, in particular, the fact that normal adult epithelial cells seem to never expose STn epitope to the bloodstream and immune system, turns it into an interesting antigen for immunization. So far, few strategies have been developed as anti-STn vaccine, such as Theratope [102,103,104,105,106] which has been pointed out for its use in clinical trials [107].

### 4.1. Theratope

The Theratope vaccine consists of a synthetic construct of STn disaccharide conjugated to the Keyhole limpet hemocyanin (KLH) that has been designed by the biotech company Biomira (now Oncothyreon, Alberta, Canada) [108]. KLH was chosen as carrier protein because of its apparent safety, while stimulating antibody production and T cell activation. In addition, KLH offers a great carrier capacity as one mole of KLH can be substituted with approximately 3000 moles of glycan hapten, which is crucial to better simulate the common high STn density on clusters of mucins found on tumor cells.

#### 4.1.1. Theratope in Clinical Trials

Theratope was initially designed for use in metastatic breast cancer. In 1996, the results of Phase-II studies in women with metastatic breast cancer were published [109,110,111,112] and abundantly reviewed some years later [104,105,106,113,114,115,116,117,118]. In these clinical trials, Theratope immunotherapy was well tolerated by vaccinated patients with minimal toxicity [110,111]. Patients receiving Theratope had a significant improved survival by 12.1 months and developed anti-STn humoral immune responses [113]. These data enlightened the relevance of this antigen as tumor-specific antigen, and its safety showed that the vaccine was unable to break immune tolerance to other self-antigens and was not generating adverse autoimmune responses. 

Patients with metastatic breast cancer receiving low-dose intravenous cyclophosphamide (inhibitor of suppressor T cells) before vaccinations showed longer survival and generated higher antibody titers than control patients [110,111]. In a single-arm Phase II study, the Theratope was used in 95 women with metastatic breast cancer undergoing therapy with aromatase inhibitors or estrogen receptor antagonists [119]. The primary objective of this trial was to determine tolerability and immune responses elicited by the vaccine in patients with hormone-sensitive breast cancer who did not require chemotherapy.

Another combined therapy was tested on patients with advanced breast or ovarian cancers. Patients were treated with high-dose chemotherapy, followed by autologous stem cell rescue to restore the immune cells, and then vaccinated with Theratope. Interestingly, the patients developed T cell responses against cancer cells, as demonstrated by the lytic activity against STn positive cancer cell lines. The cancer vaccine was well tolerated in patients after autologous transplant and, while not highly significant, the trends in data supported the concept that the Theratope vaccine decreased the risk for relapse and death [109,120].

Regarding the combination with chemotherapy, a Phase II study assessed the efficacy and safety of Theratope in patients with colorectal cancer receiving first-line chemotherapy [121]. The results showed that patients were capable of mounting an immune response to the vaccine while receiving concurrent chemotherapy. Side effects, such as mild injection site reactions with ulcerations were reported. Nevertheless, this trial was the first describing the efficacy of using the Theratope vaccine in combination with chemotherapy. Interestingly, the ability of Theratope to stimulate an immune response was not affected by chemotherapy and almost all patients showed IgG responses [121]. 

The encouraging results led to the fulfilment of a randomized, double-blind, Phase III trial across 126 centers in 10 countries, involving 1030 women with metastatic breast cancer. Nonetheless this trial failed to demonstrate that Theratope improved median time to disease progression or overall patient survival [107]. 

The potential explanation for the failure of the Phase III clinical trial is that the patient population was not evaluated for STn expression prior to enrolment, possibly masking any benefit from the vaccine due to heterogeneous STn expression between patients.

At the same time as it was concluded that the vaccine did not increase survival in patients with metastatic breast cancer disease, the analysis of a pre-stratified subset of patients who were receiving hormonal therapy showed a significant difference in median overall survival, with an increased survival by 6.5 months when treated with Theratope [122]. This was in some way unanticipated since it had been reported that STn expression was either not correlated with hormone receptor positivity [39,66] or was associated to hormonal receptor negativity [67]. Moreover, STn is associated with loss of cell differentiation, while hormonal receptor expression is known to be correlated with a differentiated state of breast tumor cells [123,124]. Thus, the reasons why Theratope seemed to be only efficient in this subset of patients remain unclear.

While additional investigations are needed to clarify the discrepant results concerning the Theratope clinical trials, the overall results point to a relative efficacy and safety of this anti-STn immunotherapy.

#### 4.1.2. Highlights on the Theratope Mechanism

With the purpose of elucidating the mechanisms involved in the Theratope efficacy, some noteworthy studies have been performed [125,126]. 

Braun *et al*. centered on the results from the Phase III clinical trial showing a prolonged survival in Theratope-vaccinated patients who were treated with concomitant hormone therapy, and they built the hypothesis that the immune response elicited in Theratope-treated patients could collaborate with the effects of hormonal therapy, improving anti-tumor responses. These authors proved that, by an undescribed mechanism, tumor cells lines when treated with aromatase inhibitors exhibit increased sensitivity to both monocyte-mediated and antibody-dependent cellular cytotoxicity. Therefore the hormone-based treatment may collaborate with anti-tumor antibodies (e.g., anti-STn antibodies induced by Theratope) to produce improved tumor control in patients [125]. 

In another study, using a murine model of breast cancer, Burchell’s group also clearly demonstrated that Theratope-induced tumor protection was dependent on the quantity of anti-STn antibodies raised by immunization [126]. Interestingly, anti-STn antibodies were able to recognize a wide range of STn-carrying glycoproteins, such as osteopontin expressed by mammary carcinomas, suggesting that a response to multi-targets expressing STn was important to induce tumor protection.

It is widely known that anti-tumor antibodies could delay tumor growth by antibody-dependent cellular cytotoxicity, inhibition of function or a combination of the two mechanisms. Pre-clinical and clinical studies all showed that immunization with Theratope usually induces STn-specific IgGs [107,118,126], including the IgG2a subtype, known to mediate antibody-dependent cellular cytotoxicity in mouse models [127]. While this could explain the observed delay in tumor growth in mice and the increased time to progression in patients [107,126], little is known about the role of the Theratope-induced antibodies in the inhibition of tumor function. However, Blixt *et al*. recently reported that the detection of high titers of auto-antibodies directed against MUC1 cancer specific glycoforms, including MUC1-STn, in early stage breast cancer patients was associated with increased time to metastasis, supporting the protective role of anti-STn antibodies [128].

### 4.2. What Can Be Improved in Anti-STn Approaches?

#### 4.2.1. Inducing Better Immune Responses

Based on the data referred to above, it is widely assumed that if a vaccine could elicit a strong immune response towards STn, it can potentially exert increased protective effects in the host against cancer. However, there are significant immunological challenges to develop an active anti-tumor immunotherapy based on STn antigens, due to its low immunogenicity [129,130].

**Figure 5:**
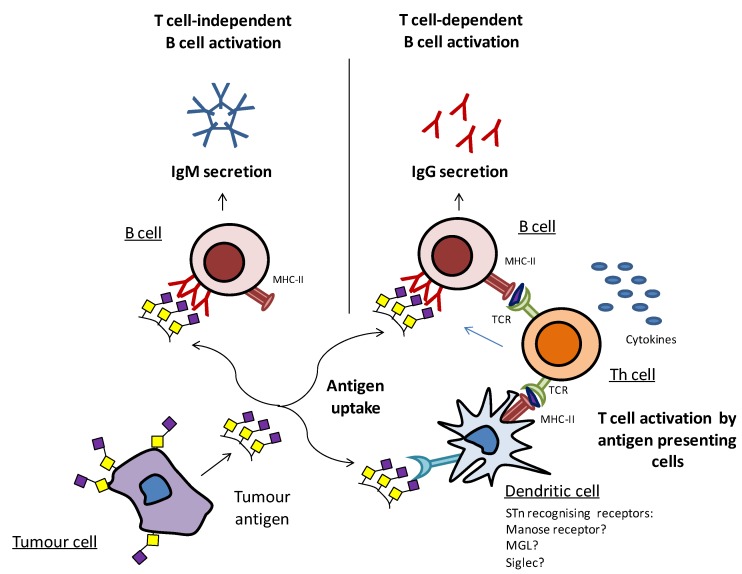
Simplified depiction of tumor antigen recognition and the induction of humoral immune responses. Tumor antigens can be directly recognized by B cells, and the cross-linking of the B cell receptors leads to IgM secretion through T cell-independent B cell activation. Tumor antigens can be endocytosed by antigen presenting cells, such as DCs and then presented to Th cells, thus leading to T cell activation and subsequent T cell-dependent B cell activation.

Tumor immunology involves two main interrelated mechanisms, the cellular immune response and the humoral response. Cell-mediated immunity involves the activation of a variety of immune cells, including antigen presenting cells, such as dendritic cells (DCs) and macrophages, which uptake antigens and then present a small portion of peptide antigens (epitopes) to activate specific CD4^+^ helper T cells (Th) and CD8^+^ cytotoxic T cells [131]. The T cell receptors (TCR) are restricted to recognizing antigenic peptides only when presented in the context of MHC, by the antigen presenting cells (Figure 5). DCs are pivotal due to their role in regulating both innate and adaptive immune responses, their migratory capacity, and by dictating whether the course of the immune responses will be tolerogenic or immunogenic [132,133]. The humoral response, in contrast, is mediated by B cells that secrete antibodies, upon cross-linking of their receptors (B cell receptors) with specific epitopes. IgM is the first immunoglobulin isotype to appear, but due to its relatively low affinity to antigens and short time period, it provides only a quick, short-term protection. Only when B cells are able to receive stimulatory signals from Th cells (T cell-dependent B cell activation), the switching of antibody subtypes from IgM to high-affinity IgGs takes place (Figure 5). These high-affinity IgG antibodies can bind with the target cancer cells, marking them for destruction by either the complement or antibody-dependent cell-mediated cytotoxicity. The T cell-dependent B cell activation is essential for the generation of significant memory B cells and for long-lasting humoral responses [131,134,135].

Carbohydrates when administered alone activate the B cells through a T cell-independent mechanism (Figure 5), as by themselves they cannot be presented by MHC and then recognized by T cells. Without additional help from Th cells, no high-affinity IgG antibodies can be generated. Yet, when carbohydrates are conjugated to a protein carrier, peptide presentation takes place, providing the activation of B cells through a T cell-dependent mechanism [136,137]. 

Thus, in order to induce T cell-dependent B cell activation, the STn antigen has been covalently linked with an immunogenic carrier, such as KLH in Theratope, which contains protein epitopes that can be presented by antigen presenting cells and be subsequently recognizable by the Th cells. Apparently and given the reported increased titers of IgG1 and IgG2a but also of IgM, it may be hypothesized that both T cell-dependent and -independent B cell activation occur during Theratope immunization. The chemical conjugation of STn with other protein or peptide carriers may probably improve the switch to T cell-dependent activation, increasing the IgG/IgM ratios, thus leading to improved anti-tumor responses. 

Notably, in the beginning of 90s, partially desialylated ovine submaxillary mucins, which contained Tn and STn antigens were used to immunize patients with colorectal cancer [138] and induced both IgM and IgG antibodies against STn, thus proving the immunogenicity of this type of conjugate. Remarkably, glycopeptides bearing multiple Tn epitopes also proved to be immunogenic, inducing anti-STn antibodies without the use of a carrier protein [139]. However, improved titers were obtained with glycopeptides bearing Tn and/or STn that were conjugated with carriers such as KLH [140,141] or tetanus toxoid [142]. However, *in vivo* studies showed that the humoral responses induced by such peptides were still not sufficient to provide protection from a tumor challenge [126]. Comparative studies evaluating the humoral responses that are elicited based on the type of STn carrier are still necessary.

Interestingly, we have found that desialylation of DCs potentiates the immune responses they triggered, in particular their unique capacity to prime Th cells [143]. Concordantly, cancer cells expressing STn antigen tend to inhibit DC maturation and hinder subsequent T cell activation, when compared with parental cells lines with absent or low expression of STn [144] suggesting that STn-expressing cancer cells are prone to cause immune tolerance. Thus, to the immunological challenge of anti-STn immunization, one should include the identification of tools to fine tune the innate response and thus surpassing tolerogenic responses. Some approaches have actually been performed in that sense, which include the concomitant use of selected adjuvants and the use of Toll like receptor agonists such as CpG and BCG. However, many lessons are still to be learnt from vaccines against other tumor associated antigens, whose results have been recently reviewed [145,146] and therefore are not included in this article. 

As referred to above, the antigen structure is crucial for tuning the immune responses during immunization and carbohydrate antigens, as the STn antigens have to be coupled to a relevant protein or peptide carrier to trigger long-lasting immune responses. The identification of the physiological STn-positive proteins present in cancer cells is another key piece to improve anti-STn immunization. On one hand, STn-positive proteins can be used as immunogen in vaccination protocols, expanding the immunization targets while providing means to foment T cell-dependent B cell activation. On the other hand, STn-positive proteins offer the possibility to target more specific pathological mechanisms. 

#### 4.2.2. Co-targeting STn and Relevant Glycoproteins

So far the available information relies on human cell lines and of the various STn-positive proteins present, four have been fully confirmed: MUC1 and CD44 in human [6,78], integrin β1 [147] and osteopontin [126] in mice. All of these molecules have described functions in adhesion and/or migration and chemotaxis, and it has been suggested that the modification of their STn content may influence their function and the mobility of the cancer cells [148]. A comprehensive compilation of our current knowledge about the role of STn-positive glycoproteins and their potential effect on tumor behavior has been recently reviewed [148]. While, the physiological role of STn on tumor cell behavior remains obscure, targeting these glycoproteins is more likely to interfere with specific mechanisms involved in tumor development. Moreover the targeting of multiple epitopes may be advantageous in overcoming the problem of tumor escape that has been documented in a number of clinical studies [149].

There has been particular interest in MUC1 as an immunotherapeutic target. MUC1 are expressed on the luminal surface of epithelial cells and act in cell adhesion and signaling [150]. However, on many carcinomas, MUC1 is over-expressed and found on all cell surfaces. Here, MUC1 also display aberrant *O*-glycosylation patterns including STn [151], causing different B and T cell-specific antigenic epitopes to be exposed. 

A limited number of MUC1-based vaccines are now being used and evaluated in advanced clinical trials. PANVAC-VF and MVA-MUC1-IL2 are virus-based vaccines expressing MUC1 and T cell co-stimulatory molecules (B7.1, intracellular adhesion molecule-1 and leukocyte function-associated antigen-3) [152] or interleukin-2 [153], respectively. Stimuvax is a liposome-based vaccine composed of a synthetic MUC1 peptide, coupled with the adjuvant monophosphoryl lipid A, evaluated in NSCLC [154]. ImMucin, which is being evaluated in multiple myeloma patients, consists of a synthetic peptide composed of the entire signal peptide domain of the MUC1 that is expressed only on tumor cells in association with MHC molecules, thereby ensuring specific anti-cancer activity [155]. In these trials, there is a clear concern not only over targeting the MUC1 tumor antigens, but also over boosting either innate and/or adaptive immune responses. However, no attention has been given to the modifications that MUC1 may have related with STn or other *O*-glycosylation types. These variables would be important to consider since specific glycoforms of MUC1, bearing Tn, STn, sialyl-Lewis^a^ and/or sialyl-Lewis^x^ antigens have been detected in different types of cancer [156,157,158,159]. Specific glycoforms of MUC1 or MUC1 glycopeptides distinctively modulate the immune response that is set up against tumors [160]. This may be due to the fact that MUC1 glycopeptides can be presented to cytotoxic T cells, and glycans are integral parts of their TCR defined epitopes [160]. However, densely glycosylated MUC1 glycopeptides are unable to be processed by antigen-presenting cells [161], compromising antigen presentation and, consequently, T cell activation. By contrast, glycopeptides carrying the Tn or TF antigens induce carbohydrate-specific cytotoxic T cell response in mice [162]. These observations reinforce the complex role of glycosylation in the modulation of cellular immune responses. Humoral responses to MUC1 have also been observed in carcinoma patients. In breast cancer patients, the presence of circulating antibodies against MUC1 at the time of cancer diagnosis has been correlated with a favorable disease outcome. Furthermore, modification of the MUC1 peptides with GalNAc (Tn antigen) leads to stronger antibody binding, probably due to the changes in conformational epitopes [128,163,164]. The interpretation of all this data in the sense of fully understanding how glycosylation modifies the immune response against MUC1 or even other proteins would lead to the development of a MUC1-based cancer vaccine that consistently elicits relevant humoral and cellular immunity that has not yet been developed.

Other approaches against MUC1, rather than molecule vaccines, are also in trials in patients with specific types of cancer. One is a novel cell-based vaccination with autologous DCs loaded with MUC1 or Tn-MUC1 peptides [155] and the other approach is the use of therapeutic humanized anti-MUC1 antibodies, such as the PankoMab-GEX from Glycotope and the radiolabeled anti-MUC1 humanized antibody, 90Y-hPAM4 from Immunomedics. Both the use of DC-based vaccines and therapeutic antibodies are excellent approaches to be considered in the future for specific anti-STn therapy. 

For the anti-STn therapy to reach the level of application that anti-MUC1 has reached, its effect on immune responses should be fully understood. This should be combined with the knowledge of the role that the STn antigen plays in the function of the STn-expressing proteins, MUC1 and others and in overall tumor cell progression in order to identify potential synergistic solutions for treating STn-expressing carcinomas.

#### 4.2.3. Combining STn with other Cancer Associated Carbohydrates

Considering that beyond STn other tumor associated carbohydrate antigens are usually aberrantly expressed by cancer cells [165], multi-carbohydrate antigens covering different carbohydrates found aberrantly expressed in cancer cells have been constructed to trigger multi-antigenic responses in patients. Glycopeptide dendrimers have therefore been developed to simultaneously present different carbohydrate antigens such as STn, Tn, T, Globo H, G_M2_, Lewis^y^, and MUC1-Tn antigens. Presently, the challenge of efficiently preparing carbohydrate clusters was solved at the level of chemical synthesis [166]. These constructs and their application in pre-clinical and clinical trials have been reviewed elsewhere [167,168]. It is anticipated that these constructs will foster new platforms for effective and selective delivery of anti-tumor therapeutics.

#### 4.2.4. Whom to Treat with Anti-STn Vaccine?

As referred to above, a potential explanation for the Theratope failure in the Phase III clinical trial in breast cancer patients was the fact that the patient population was not evaluated for STn expression prior to enrolment. Just over 50% of the breast cancer cases express STn (Figure 2, Figure 3 and Figure 4). The lack of patient selection was therefore possibly masking any benefit from the vaccine due to heterogeneous STn expression between patients. 

At the same time, Figure 2 and Figure 4 show the percentages of cases expressing STn in different types of cancer and reveal different populations that may benefit from STn-targeting therapies, such as the already mentioned patients with breast cancer, colorectal cancer as well as patients with gastric, ovarian and pancreatic cancer. In fact, the STn neo-expression or over-expression was reported with highest frequencies in pancreas, colorectal and ovarian cancers. However, considering that the role of STn may differ from cancer to cancer and probably from individual to individual, it is likely that selective approaches should be developed and patients should not be treated exactly by the same STn-targeting therapy, but instead based on their personal tumor profile.

On the other hand, STn immunotherapy has always been tested as a post-surgical adjuvant therapy in combination with hormonotherapy or chemotherapy, with the aim of improving survival by decreasing relapse, as discussed above. However, recent data in the literature suggest that anti-cancer vaccine might be more efficiently used in a prophylactic rather than in a therapeutic way [169,170]. Interestingly, anti-STn antibodies were detected in healthy women who never developed cancer in the 25 to 30 years following the date of blood sampling [128]. Assuming that STn presentation to the immune system has to result from carcinogenesis, the authors propose that these anti-STn antibodies might be functional in suppressing tumor development and progression. This concept is further supported by a recent study showing that a robust and specific auto-immune response against a cancer specific antigen is able to prevent the emergence of autochthonous ovarian tumors and control the growth of established ovarian malignancies [171].

**Figure 6:**
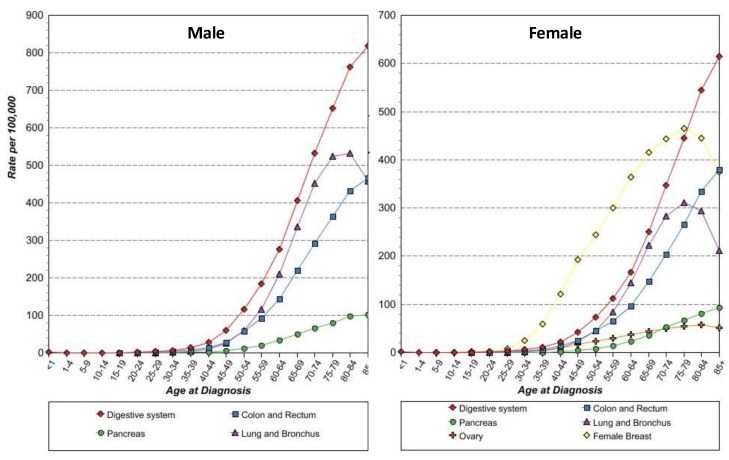
Age-specific (crude) SEER incidence rate by cancer sites. All ages, all races, 1992-2009.

With these concepts in mind, anti-STn vaccine, such as Theratope, may become attractive again, now as a prophylactic anti-cancer vaccine, for several reasons. First, the protective effect of anti-STn has already been demonstrated *in vivo*. Second, the efficacy of the anti-STn vaccine to induce a proper immune response in humans without major risks of autoimmune responses in patients has been reported repeatedly. Third, there is a substantial body of evidence showing that STn is expressed in early carcinogenesis in all the epithelial cancers investigated (Figure 4) making these cancers good targets from the earliest stage of the tumors. At last, most of these cancers where STn is more frequently expressed, such as cancers of the digestive tract, pancreas, colon, lungs and ovaries, are also cancers whose incidence are associated with age, with a drastic increase from 45 years onwards (Figure 6). Thus the whole population above 45 years of age would constitute a suitable cohort to test the ability of anti-cancer vaccine to decrease epithelial cancer incidence. 

There are some prophylactic vaccines being tested for their ability to decrease cancer incidence by protecting the patients against potentially cancer-inducing viral infection. For example, anti-papilloma virus vaccines have been shown to protect the immunized population against cervical cancer [172] However, it is noteworthy to know that prophylactic anti-cancer vaccines directly targeting a cancer-associated antigen have never been tested in clinical trials [169]. For the reason listed above, we believe that an improved anti-STn vaccine should be a good candidate to be among the first to be tested in the future.

## 5. General Conclusion

While additional investigations are needed to clarify the discrepant results concerning Theratope clinical trials, the pursuit of improved anti-STn immunotherapy remains an active area of investigation. The overall concept of anti-STn vaccines as any other anti-cancer vaccine is a promising idea. However, putting the theory into practice has proven to be challenging. The advances in vaccines using STn-expressing proteins, other carbohydrate antigens and DC-based vaccines associated with a robust knowledge of the STn physiological function and the identification of means of surpassing tolerogenic responses will warrant a selective and successful development of anti-STn therapy, possibly in a prophylactic setting. Other anti-STn therapeutic approaches, such as therapeutic antibodies that may prove particularly useful in targeting immune responses and also drug delivery are an expected open field.
